# Continuous Glucose Monitoring and Exercise in Type 1 Diabetes: Past, Present and Future

**DOI:** 10.3390/bios8030073

**Published:** 2018-08-03

**Authors:** Shaelyn K. Houlder, Jane E. Yardley

**Affiliations:** 1 Augustana Faculty, University of Alberta, 4901-46 Ave, Camrose, AB T4V 2R3, Canada; shoulder@ualberta.ca; 2 Alberta Diabetes Institute, 112 St. NW, Edmonton, AB T6G 2T9, Canada

**Keywords:** exercise, hypoglycemia, hyperglycemia

## Abstract

Prior to the widespread use of continuous glucose monitoring (CGM), knowledge of the effects of exercise in type 1 diabetes (T1D) was limited to the exercise period, with few studies having the budget or capacity to monitor participants overnight. Recently, CGM has become a staple of many exercise studies, allowing researchers to observe the otherwise elusive late post-exercise period. We performed a strategic search using PubMed and Academic Search Complete. Studies were included if they involved adults with T1D performing exercise or physical activity, had a sample size greater than 5, and involved the use of CGM. Upon completion of the search protocol, 26 articles were reviewed for inclusion. While outcomes have been variable, CGM use in exercise studies has allowed the assessment of post-exercise (especially nocturnal) trends for different exercise modalities in individuals with T1D. Sensor accuracy is currently considered adequate for exercise, which has been crucial to developing closed-loop and artificial pancreas systems. Until these systems are perfected, CGM continues to provide information about late post-exercise responses, to assist T1D patients in managing their glucose, and to be useful as a tool for teaching individuals with T1D about exercise.

## 1. Introduction

Physical activity and exercise are a challenge to maintaining blood glucose in individuals with T1D. Since the 1980s, laboratory and field-testing sessions have been used to elucidate the diverse blood glucose responses to different types and durations of activity in this population. Researchers now know that aerobic activities of various intensities and durations lead to declines in blood glucose and a high risk of hypoglycemia [1,2,3,4]—unless these activities are performed in a fasting state first thing in the morning, where increases in blood glucose have been observed [5,6]. A similar trend has been found for resistance exercise, where studies performed in the afternoon observed declines in blood glucose [7,8], while those performed in the morning were associated with either an increase [9,10], or no effect [11] on blood glucose concentration.

Studies have also shown that high intensity (anaerobic) activities have the opposite effect on blood glucose to aerobic activities: when performed in short duration, they lead to an increase in blood glucose [12,13], and the potential for post-exercise hyperglycemia in individuals with T1D [12,14]. This phenomenon has been harnessed in the form of short sprints (either before [15] or after exercise [16,17]) and intermittent high intensity exercise protocols [4,18,19,20,21], that have been shown to protect against declines in blood glucose concentration when compared to performing aerobic exercise alone, in spite of more energy being expended. The physiology behind blood glucose changes during different types, intensities, and timings of exercise are reviewed in detail elsewhere [22,23,24,25,26].

While most of these trends in blood glucose were discerned before the widespread use of continuous glucose monitors (CGM), it is what this technology has enabled researchers to discover in terms of post-exercise (especially nocturnal) hypoglycemia that can be considered the most useful and relevant to patient safety. As real-time CGM becomes more affordable and accessible to patients, health care providers are becoming able to better tailor approaches to exercise and physical activity based on individual responses. With every improvement in CGM and sensor technology, the research community comes closer to developing an artificial pancreas that will be able to manage physical activity and exercise in T1D patients.

This review aims to examine the current state of knowledge related to T1D and physical activity/exercise in adults as a result of the use of CGM in research. It will also discuss the potential of using CGM as a teaching tool for training patients on blood glucose management during different types of physical activity. Finally, it will discuss how improvements in CGM technology have made the development of closed-loop systems possible, and how this technology is being developed to suit the context of physical activity and exercise in T1D.

## 2. Materials and Methods

**Figure 1:**
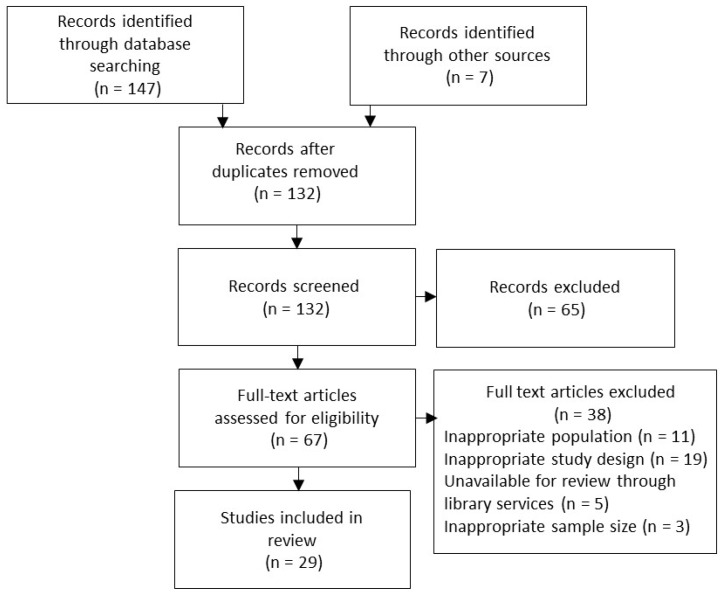
Search strategy flow chart.

A strategic search was completed using the PubMed and Academic Search Complete databases employing the search terms “type 1 diabetes”, “T1D”, “insulin dependent diabetes mellitus”, “IDDM”, “juvenile diabetes”, “exercise or physical activity”, “continuous glucose monitoring”, and “sensor augmented pump or sensor augmented insulin pump or SAP”. Duplicate articles were removed, and the remaining articles were reviewed, first through assessing the title and abstract, and then more thoroughly by evaluating the full text after initial articles deemed inapplicable were removed. Figure 1 illustrates the procedure implemented to obtain literature. Studies were selected based on sample size and participant age. Studies including small sample sizes (n < 5), adolescents, pregnant women, individuals with type 2 diabetes, or which were described as “quasi experimental” were excluded. Upon completion of the search protocol, 29 articles were reviewed. 

## 3. Discussion

### 3.1. CGM-Derived Contributions to the T1D Exercise Research Literature

Until recently, few studies have been able to observe the post-exercise trends in blood glucose levels in adults with T1D. Prior to the era of CGM, such studies were prohibitively expensive and time demanding for both participants and researchers, as they often involved an overnight stay in a hospital or lab setting, with frequent blood sampling. More recently, long periods of observation post-exercise have enabled the detection of exercise modality-specific blood glucose trends, albeit in the presence of substantial variability.

#### 3.1.1. Aerobic Exercise

Most research surrounding exercise in individuals with T1D has been focused on continuous aerobic exercise, or comparing other forms of exercise to this modality. Aerobic exercise involves the use of systems that produce energy aerobically to fuel activity, and consists of the prolonged (more than 10 min), rhythmic, and repetitive use of large muscle groups (e.g., walking, jogging, cycling, swimming, etc.) [27]. This type of exercise is known to strengthen the lungs and the cardiovascular system, improve mental agility, shorten healing time [28], decrease resting systolic and diastolic blood pressure [29], and reduce stiffness in central arteries [29].

In using CGM to observe blood glucose after aerobic exercise, studies have found that blood glucose can increase and remain elevated for several hours post-exercise [7,20,30]. When exercise is performed in the afternoon, however, declining blood glucose levels several hours after exercise can lead to an elevated risk of nocturnal hypoglycemia. Maran et al. [31] reported that 30 min of moderate aerobic exercise at 40% of maximal aerobic capacity (VO_2_max) resulted in increasing blood glucose levels for up to two hours following exercise and decreasing blood glucose levels beginning 4 h post-exercise, indicating a delayed risk of hypoglycemia. Iscoe and Riddell (2011) also found a decrease in interstitial glucose, as measured by CGM, approximately 4–7 h after 45 min of aerobic exercise (55% of peak work rate), when it was performed late in the afternoon [20]. A similar intensity (60% VO_2_max) and duration of aerobic exercise performed at 4 pm produced hypoglycemic events (captured by CGM) on 65% of the nights following aerobic exercise, with 3.7 ± 8.4% of the time during the night following aerobic exercise being spent with interstitial glucose levels <3.0 mmol/L [32]. Thus the use of CGM has been instrumental in gleaning information about the frequency, severity. and timing of hypoglycemia after a bout of aerobic exercise in individuals with T1D.

Within the context of aerobic exercise, CGM has also been used to demonstrate that food and supplement intake will have an impact on blood glucose levels following activity [33]. A double-blind, placebo-controlled study examining the effect of caffeine ingestion on glycemic control during moderate aerobic exercise (45 min at 60–70% of VO_2_max) found a correlation between caffeine intake (6 mg/kg of body weight) and higher blood glucose levels before bed [33]. This protective effect was short-lived, however, as CGM data also revealed lower blood glucose levels in the morning after the exercise session that involved caffeine ingestion versus one where a placebo was consumed [33]. Studies examining insulin and carbohydrate adjustments for aerobic exercise are discussed in more detail below.

#### 3.1.2. High Intensity Intermittent Exercise

By using CGM, researchers have become increasingly aware that post-exercise responses to high-intensity interval exercise (HIIE), a type of aerobic exercise that involves continuous exercise with short duration, high-intensity bouts spaced throughout, are extremely variable. To date, one study shows a decrease in the risk of nocturnal hypoglycemia post-exercise [20], while another shows no impact [4], and two show an increase in risk [31,34]. The HIIE study showing a protective effect (i.e., higher overnight interstitial glucose compared to aerobic exercise) involved cycling at 50% of peak work rate with 15-s sprints every 5 min, performed at 5 pm [20]. In spite of the early protection against hypoglycemia, a sharp decline in interstitial glucose was measured at 6 am (approximately 11 h post-exercise), indicating that HIIE may delay hypoglycemia rather than prevent it [20]. In a separate HIIE study, participants with T1D attended a 60-min spin class designed to keep participants’ heart rates at about 60% of their respective maxima (~40% VO_2_max). During the class, frequent changes were made to resistance and cadence, resulting in variable intensity throughout. All participants experienced a hypoglycemic event within 22 h of exercise [34]. While these two studies involved exercise at 5 pm, performing HIIE (30 min of cycling at 40% of VO_2_ max with 5-s sprints every two minutes) earlier in the afternoon (2 pm) in a different study simply shifted the declines in blood glucose back a few hours, with CGM detecting the lowest interstitial glucose concentrations between midnight and 6 am [31]. These CGM-based findings have important implications for patient safety, as knowing the window of highest hypoglycemia risk for HIIE is different from that of aerobic exercise allows patients to adjust insulin dosage and carbohydrate intake accordingly for each type of activity.

#### 3.1.3. Resistance Exercise

Resistance exercise is an activity performed to improve muscle strength, endurance, and power [35]. It involves muscular work against a form of resistance such as a weight or elastic resistance band. What is currently known about the post-resistance exercise blood glucose trends of individuals with T1D was gleaned from a small number of studies using CGM. Compared to aerobic exercise (45 min at 60% of VO_2_max) at 5 pm, resistance exercise (3 sets of 8 repetitions at the participants’ 8 repetition maximum) led to lower interstitial glucose levels in the fourth and fifth hour following exercise. More frequent but mild nocturnal hypoglycemia was also found following resistance exercise in comparison to aerobic exercise [7]. Reddy et al. [32] had similar findings using an almost identical resistance exercise protocol, with 70% of nights having at least one hypoglycemic event. Time spent with interstitial glucose <3 mmol/L during the night was 1.8 ± 7.3%. Taken together, these studies indicate that resistance exercise increases the likelihood of hypoglycemia; however, the severity of hypoglycemia (when it occurs) may be greater after aerobic exercise [32].

When resistance and aerobic exercise are performed in sequence by individuals with T1D, nocturnal blood glucose trends may differ based on which modality was performed first [8]. While no significant difference was found in the frequency of nocturnal hypoglycemia between protocols (resistance then aerobic exercise vs. aerobic then resistance exercise), performance of aerobic exercise first resulted in a trend (*p* = 0.06) towards increased duration and depth of hypoglycemia during the night, as measured by CGM (area under the curve) [8]. Armed with this information, individuals with T1D are able to change how they combine their exercises to cater to whichever difficulty they face most frequently (hypoglycemia or hyperglycemia).

### 3.2. CGM Accuracy during Exercise

While CGM provides a cost effective [36] way to continuously monitor interstitial glucose levels of patients in a practical or research setting [37], its accuracy during various forms of exercise has occasionally been questioned, and is thus still under investigation [38,39,40,41,42]. With improvements in sensor technology and device-related algorithms, CGM systems have rapidly improved their accuracy over the past decade. During that time, studies have assessed CGM accuracy during continuous aerobic [39,40,41,42], high intensity interval [39,40], and resistance exercise [42]. 

Some of the earlier studies examining CGM accuracy during exercise found a tendency for CGM to differ from venous blood glucose levels, a phenomenon which was often attributed to sensor lag. A 2012 study [41] testing CGM accuracy of the Medtronic Guardian Real-Time system (Medtronic Mini-Med, Northridge, CA, USA) during 30 min of moderate to high intensity exercise found clinically acceptable CGM accuracy (correlation of 0.957) during the most strenuous of three aerobic exercise intensities performed by the T1D participants, where the authors were expecting the lowest accuracy. During the same study, the mean absolute difference between CGM measurements and venous blood glucose was measured at 0.56 ± 1.72 mmol/L, with CGM overestimating blood glucose [41]. A separate study comparing sensor (Medtronic Sofsensor) performance of the CGMS System Gold (Medtronic, Northridge, CA, USA) blinded CGM during 45 min of aerobic (treadmill running at 60% VO_2peak_) or resistance exercise revealed an underestimation of blood glucose which was greatest during resistance exercise (median absolute difference of −1.9 mmol/L, −0.6 mmol/L, and 0.3 mmol/L during hyperglycemia, euglycemia, and hypoglycemia respectively) and smallest during aerobic exercise [42]. In spite of these differences, sensor accuracy was deemed to be adequate during all types of exercise [42].

A CGM accuracy test published in 2016 [39] compared Dexcom G4 Platinum (Dexcom, San Diego, CA, USA) sensor performance during continuous exercise (90 min of cycling at 50% VO_2_max) to its performance during HIIE (90 min of cycling at 50% VO_2_max with 10 s maximal sprints every 10 min) performed by individuals with T1D. Although HIIE and continuous aerobic exercise result in significantly different blood glucose values and trends, there was no significant difference between the two sessions with respect to the accuracy of the CGM when compared to venous blood glucose (Mean absolute relative difference (MARD) 13.3 ± 2.2% and 13.6 ± 2.8% for HIIE and continuous exercise respectively) [39]. 

Another study, published in 2016 [40], compared the accuracy of the Medtronic Guardian Real-Time system using Enlite sensors (Medtronic Diabetes, Northridge, CA, USA) during three different aerobic activity and HIIE levels. Continuous glucose monitoring was shown to overestimate capillary glucose values during low, moderate, and higher intensity aerobic exercise (5% below and above the first lactate turn point and 5% below the second lactate turn point), and for three separate HIIE protocols (20 s maximal sprints every 120, 60, or 20 s for a total of 30 min, with the intensity between intervals being identical to each of the three aerobic exercise protocols) performed by individuals with T1D. A significant difference in CGM accuracy was found in continuous exercise versus HIIE for all intensities [40]. Mean absolute relative difference, between continuous and high intensity interval exercise was 19.8 ± 14.5% vs. 16.9 ± 9.1% (*p* = 0.13) for the low intensity vs. low intensity with HIIE, 12.8 ± 8.2% vs. 26.5 ± 17.6 (*p* < 0.0001) for the moderate intensity vs. moderate intensity with HIIE, and 23.7 ± 10.8% vs. 15.5 ± 10.8% (*p* = 0.001) for the high intensity trial vs. high intensity with HIIE. In spite of the differences in performance of GCM during different modalities and intensities, the accuracy of the sensors during both types of exercise was clinically acceptable according to the Clarke error grid [40]. During continuous exercise, the correlations were 0.93, 0.92, and 0.96 for light, moderate, and high intensity respectively, while they were 0.74, 0.99, and 0.91 during HIIE [40]. 

More recently (2017), a study comparing three different CGM devices [38]—Abbott FreeStyle Libre (Abbott Diabetes Care, Alameda, CA, USA), Dexcom G4 Platinum (Dexcom, San Diego, CA, USA), and Medtronic MiniMed 640G (Medtronic, Northridge, CA, USA)—during moderate aerobic activity (50% of VO_2_max), performed by individuals with T1D, both before and after a meal, found a high level of accuracy in all three devices during exercise. The Abbott system was reported to have the best accuracy, with the lowest MARD (13.2 ± 10.9%) in comparison to the Dexcom (16.8 ± 12.3%) and the Medtronic (21.4 ± 17.6) systems [38]. It was noted, however, that CGM performance was slightly lower when participants were experiencing hypoglycemia [38]. Overall, recent studies seem to agree that, in spite of small differences between CGM readings and venous glucose, current CGM sensors are performing at an adequate level for reflecting changes in blood glucose during exercise, and are thus useful tools for patient safety both during and after exercise.

### 3.3. CGM and Diabetes Management during Exercise

In addition to providing greatly-needed information about post-exercise blood glucose trends associated with different types and timings of exercise, CGM technology has also been used to further investigate the impact of various insulin adjustments prior to aerobic exercise [43,44,45,46,47]. In individuals with T1D treated by multiple daily insulin injections (MDI), reduction of pre-exercise and post-exercise insulin doses by 75% and 50% respectively was found to maintain glucose levels during exercise (45 min of running at ~70% VO_2_max), and prevent hypoglycemia in the first 8 h following exercise. Despite this early protective effect, the 50% post-exercise insulin reduction was not found to protect against late onset hypoglycemia, with blood glucose responses 8 h post-exercise becoming similar to the two other treatments of a 0% and 25% reduction of insulin post exercise [43]. There was no significant difference in the frequency of late-onset (8 h post-exercise) hypoglycemia, as measured by CGM, between the treatments [43].

Studies involving CGM have also shown that while insulin adjustment may be a useful tool for managing glycemia, it may result in an unwanted increase in hyperglycemia unless carbohydrate intake is also adequately managed. A study by Campbell et al. [44] showed that combining insulin adjustments (75% decrease in pre-exercise bolus) with intake of low glycemic index carbohydrate eaten as a meal and bedtime snack after exercise (45 min at 70% of VO_2_peak) results in less post-prandial hyperglycemia (compared to a high glycemic index meal), while also providing protection against hypoglycemia. In spite of improved blood glucose outcomes before, during, and shortly after exercise, however, CGM measurements detected a persistent late-exercise hypoglycemia risk [44].

Insulin adjustments using insulin pumps have also been examined in this manner. Prevention of hypoglycemia, as measured by CGM, was noted when the basal rate in insulin pump users was reduced by at least 80%, beginning at the start of exercise and lasting two hours following a 30-min aerobic exercise protocol (75% of VO_2peak_) performed 3 h after lunch [45]. The same study also used CGM to demonstrate that a more modest adjustment of between 50% and 80% was sufficient to protect against hypoglycemia for exercise of slightly lower intensity (50% of VO_2peak_) performed at the same time of day [45]. The timing of this adjustment may, however, be insufficient for exercise of more than 30 min’ duration. Zaharieva et al. [47] found that full suspension of basal insulin at the initiation of 40 min of aerobic exercise (40–50% VO_2_max) was inadequate in defending against both declines in blood glucose during exercise, and subsequent post-exercise hypoglycemia [47]. On the strength of the CGM data provided in these studies, it is recommended that basal insulin be decreased at least 60 min before exercise [24], as immediate suspension of basal insulin at the initiation of exercise may not always be an effective strategy for maintaining blood glucose levels [47]. As part of the same study, basal insulin suspension during circuit training (which counts as HIIE) resulted in less variability in interstitial glucose during the recovery period, and less time spent in hypoglycemia [47].

While CGM has provided extensive information about post-exercise trends in blood glucose for individuals with T1D, a “one size fits all” approach to exercise and physical activity will never be possible, due to the great deal of variability in blood glucose trends. It is important to note that all of the above studies were performed using blinded CGM, in order to observe the “normal” behaviors of participants using standard tools of blood glucose management. During and after exercise is, nonetheless, where real-time CGM becomes most useful for patients, as current systems are equipped with alarms to alert the wearer of either rapid declines in blood glucose, or blood glucose levels that are approaching hypoglycemia. Although “alarm fatigue” is identified as a patient concern in the use of these devices [48], there is also evidence that patients feel more confident to exercise [48,49], and have improved blood glucose management [50] when this tool is at their disposal.

A step up from simple CGM use was recently demonstrated in a study by Breton et al. [51], where CGM data were used to provide patients with advice regarding insulin and carbohydrate intake around exercise. A CGM-based decision support system (DSS) consisting of a CGM-informed bolus advisor, an exercise advisor and a retrospective insulin titration tool was tested in the context of an exercise protocol consisting of 3 × 15 min of “mild to moderate” exercise with 5 min recovery in between [51]. Compared to usual care, use of the DSS led to an improvement in the time spent below 3.9 mmol/L (from 3.8 ± 4.6% to 1.8 ± 2%, *p* = 0.018) in spite of similar pre- and post-exercise blood glucose levels [51]. Pre-exercise carbohydrate consumption was reduced (*p* = 0.003) as was the amount of rescue carbohydrate required (*p* = 0.026) and glycemic variability (*p* = 0.045). Overall, this combination of CGM and DSS could be a safe and feasible method of improving exercise safety in individuals with T1D.

### 3.4. Using CGM as a Tool for Patients to Learn about Exercise

Another option that has been explored is the use of CGM as a teaching tool in the context of exercise for both health care providers and individuals with T1D. Using a combination of qualitative and quantitative surveys, Dyck et al. [49] ascertained that, of all of the tools used in a boot camp setting (in-class instruction, real-time CGM, supervised exercise), real-time CGM (Dexcom G4 Platinum, Dexcom, San Diego, CA, USA) was considered the most useful by the participants (a combination of health care providers and T1D patients) in learning about blood glucose responses to exercise. Most importantly, participants with T1D expressed that having the CGM improved their blood glucose control during exercise, as seeing trend arrows allowed them to better gauge whether or not carbohydrate intake was required. Participants felt that this knowledge decreased the number of times that they consumed carbohydrates unnecessarily. During qualitative interviews, some participants noted that they were less afraid to exercise, as the CGM would alert them of impending hypoglycemia. As fear of hypoglycemia is listed as one of the greatest barriers to exercise and physical activity in individuals with T1D [52], more widespread use of GCM among patients could potentially improve physical activity levels and overall health in this population. 

### 3.5. Where CGM Technology Is Leading

Improvements in CGM sensor accuracy have enabled the development of sensor-augmented insulin pumps and closed-loop systems. While CGM sensor performance during exercise is now at an acceptable level, there are still some concerns as to whether enough is known about the variability in exercise responses for closed-loop systems to adequately anticipate changes in blood glucose [53,54,55]. Recent studies would indicate that the technology is moving in the right direction. 

Abraham et al. [56] showed that sensor-augmented pump therapy could potentially be improved by adding a predictive low glucose management system that would suspend basal insulin when hypoglycemia was predicted. Following 60 min of moderate intensity (55% VO_2_max) exercise, performed in two bouts with 30 min rest in between, only 6 out of 19 participants required treatment for hypoglycemia when using the predictive low glucose management system, compared with 17 participants using standard sensor-augmented pump therapy [56]. The predictive algorithm reduced hypoglycemia, but did not reach the ultimate goal of preventing it altogether.

It has been suggested that a single-hormone closed-loop system that responds only to changes in blood glucose during exercise may respond too slowly to prevent hypoglycemia. The inclusion of additional data to control systems has resulted in improved outcomes. For example, a 2014 study by Breton et al. [57] found a significantly smaller decline in blood glucose during exercise (*p* = 0.022), but similar time spent in range when heart rate was used to inform a control to range closed-loop system. Similarly, the addition of energy expenditure (measured by accelerometry) and galvanic skin response to a closed-loop system eliminated hypoglycemia, as insulin dosage decreased during exercise, even in the presence of increasing blood glucose levels during high intensity exercise [58].

A recent study by Jayawardene et al. [59] compared the performance of a closed-loop system (Minimed 670G with Guardian Sensor 3, Medtronic, Northridge, CA, USA) in response to moderate aerobic exercise (70% of anaerobic threshold) and HIIE (six 4-min intervals between anaerobic threshold and VO_2_max with 2-min rests) in individuals with T1D. There was no significant difference between the percentage of time spent in euglycemia before, during, and after HIIE and the same time points with continuous exercise [59]. Significant differences between blood glucose levels 60 min after exercise (HIIE: 11.3 ± 0.5 mmol/L vs. aerobic exercise 8.9 ± 0.8 mmol/L; *p* < 0.001), however, illustrate that, in this particular case, there was still room for improvement in the closed-loop system’s ability to regulate blood glucose during and after different exercise modalities [59]. 

It has been suggested that for complete prevention of exercise-induced hypoglycemia, bihormonal systems, involving both insulin and its antagonist, glucagon, may be more successful, as they would better replicate the hormonal changes that usually take place [60,61,62,63]. Taleb et al. [62] compared the performance of dual hormone and single hormone systems in relation to a bout of aerobic exercise (60 min of cycling at 60% of VO_2_max) and HIIE (40 min of alternating intensities (50% and 85% VO_2_max) every two minutes with a 10-min warm-up and cool-down) [62]. Exercise was announced to the system 20 min before it began [62]. During continuous exercise, there was a trend (*p* = 0.07) for fewer participants spending time below 4 mmol/L following aerobic exercise using the dual hormone system, with 52.9% of participants experiencing hypoglycemia using the single hormone system, versus 17.6% of participants during the use of the dual hormone system [62]. During and after HIIE, the dual hormone system was also a trend (*p* = 0.07) towards significantly lower numbers of participants experiencing hypoglycemia (6.25% for dual vs. 40% for single hormone; *p* = 0.07), a decrease in the percentage of time spent in hypoglycemia [median ± IQR: 0 (0–0)% for dual vs. 22.5 (0–48.3%) for single; *p* = 0.006), and an increase the mean amount of time spent in the target range during the night following HIIE in comparison to the single hormone system (77.4 ± 15.7% for dual vs. 52.2 ± 31.7% for single hormone; *p* = 0.03) [62]. Similarly, Castle et al. found a lower percent of time spent in hypoglycemia during aerobic exercise (45 min at 60% VO_2_max) using a dual hormone system (3.4 ± 4.5%) in comparison to a single hormone closed-loop system (8.3 ± 12.6; *p* = 0.009) and also compared to a predictive low glucose suspend system (7.6 ± 8.0%; *p* = 0.001) [63]. In addition, where moderate aerobic exercise (75% of maximal heart rate) is concerned, dual hormone systems perform equally well as both open-loop and closed-loop systems in terms of maintaining blood glucose on target and preventing hypoglycemia [61].

Taking it one step further, Jacobs et al. [60] investigated the adjustment of insulin and glucagon in a dual hormone, closed-loop system in response to moderate intensity aerobic exercise (45 min at 60% of maximal heart rate) under three distinct treatments: closed-loop with insulin and glucagon adjustment, closed-loop without adjustment, and a sensor augmented pump (control). In the adjustment trial, insulin delivery was stopped for 30 min when exercise began and reduced to 50% for the hour following exercise. Glucagon delivery was doubled for 90 min beginning at the initiation of exercise [60]. The time spent in hypoglycemia after the start of exercise was lower for the adjusted system (0.3[−0.1, 0.7]%) compared to the non-adjusted system (3.1[0.8, 5.3]%), highlighting the importance of having an artificial pancreas system that adjusts dosing for exercise, and not simply in response to changes in blood glucose.

## 4. Conclusions

While CGM is a relatively new technology, implementation of the technology in a research and practical setting has improved our level of knowledge with respect to post-exercise blood glucose responses. This technology has also enabled the development of protocols for insulin and diet adjustments that help individuals with T1D manage blood glucose concentrations during and after exercise. Finally, the improvement of CGM technology has been essential for the development of closed-loop systems, which are improving in their ability to adapt to the acute stress of exercise. The continued use of CGM in further research will undoubtedly allow for the development of further management options, improving overall patient safety and confidence in undertaking exercise.
